# Corticobulbar Tract Injury, Oromotor Impairment and Language Plasticity in Adolescents Born Preterm

**DOI:** 10.3389/fnhum.2019.00045

**Published:** 2019-02-19

**Authors:** Gemma B. Northam, Angela T. Morgan, Sophie Fitzsimmons, Torsten Baldeweg, Frédérique J. Liégeois

**Affiliations:** ^1^Great Ormond Street Hospital for Children NHS Trust, UCL Great Ormond Street Institute of Child Health, London, United Kingdom; ^2^Murdoch Children’s Research Institute, Melbourne, VIC, Australia

**Keywords:** preterm birth, oromotor control, tractography, MRI, language lateralization

## Abstract

Children born preterm are at risk of impairments in oromotor control, with implications for early feeding and speech development. In this study, we aimed to identify (a) neuroanatomical markers of persistent oromotor deficits using diffusion-weighted imaging (DWI) tractography and (b) evidence of compensatory neuroplasticity using functional MRI (fMRI) during a language production task. In a cross-sectional study of 36 adolescents born very preterm (<33 weeks’ gestation) we identified persistent difficulties in oromotor control in 31% of cases, but no clinical diagnoses of speech-sound disorder (e.g., dysarthria, dyspraxia). We used DWI-tractography to examine the microstructure (fractional anisotropy, FA) of the corticospinal and corticobulbar tracts. Compared to the unimpaired group, the oromotor-impaired group showed (i) reduced FA within the dorsal portion of the left corticobulbar tract (containing fibres associated with movements of the lips, tongue, and larynx) and (ii) greater recruitment of right hemisphere language regions on fMRI. We conclude that, despite the development of apparently normal everyday speech, early injury to the corticobulbar tract leads to persistent subclinical problems with voluntary control of the face, lips, jaw, and tongue. Furthermore, we speculate that early speech problems may be ameliorated by cerebral plasticity – in particular, recruitment of right hemisphere language areas.

## Introduction

Our understanding of the impact of preterm birth on the cerebral white matter has been furthered by diffusion-weighted imaging (DWI) (Ment et al., 2009; Pandit et al., 2013; Travis et al., 2015a; Pecheva et al., 2017). Furthermore, altered diffusion metrics in specific white matter tracts have been demonstrated in relation to long-term cognitive outcome (Northam et al., 2012b; Travis et al., 2015b, 2016) and neurological function (see Ment et al., 2009 for a review). Non-speech oromotor control is known to be impaired in this population, including problems with sucking, weaning to solid food (Slattery et al., 2012; Sanchez et al., 2017) and eating difficulties persisting up to the age of 6 years (Samara et al., 2010). However, the specific neural correlates of oromotor control have rarely been examined, and studies have mainly relied on gross clinical MRI measures in younger populations (Sanchez et al., 2017).

A large proportion of children with prematurity-related brain injury have long-lasting oromotor difficulties (Northam et al., 2012a). These children struggle with the deliberate control of the jaw, lips, face, and tongue during the speech and non-speech movements. We have demonstrated that, on DWI, this impaired subgroup shows reduced fractional anisotropy (FA, a measure of water diffusivity) in the posterior limb of the internal capsule of the left cerebral hemisphere. This suggests motor tract involvement but does not identify which specific pathways have been compromised. We previously found that left corticobulbar tract injury (specifically the dorsal portion, which contributes to the control of the lips, tongue, and larynx) predicted dysarthria and oromotor dysfunction after traumatic brain injury in childhood (Liegeois et al., 2013). We therefore applied the same diffusion tractography method in the previously reported group of adolescents born preterm, who are at risk of bilateral periventricular white matter injury (Guo et al., 2017), and hypothesised that persistent oromotor impairment would be associated with reduced FA in the left corticobulbar tract.

Despite relatively normal everyday speech in adolescence, abnormal early speech development (e.g., late onset, poor speech quality) was reported by parents in many of the children with oromotor impairments – and these children were also more likely to have received speech and language therapy (Northam et al., 2012a). Given the early timing of the injury there should be considerable potential for cerebral plasticity (Jacola et al., 2006; Tillema et al., 2008; Staudt, 2010) and we therefore hypothesized that the absence of an overt speech disorder might reflect reorganization of language to the right cerebral hemisphere. We used functional MRI (fMRI) during a verb generation task to estimate the degree of interhemispheric reorganization in language-associated frontal and temporal brain regions. Having already reported language outcomes in relation to MRI/DWI findings in this cohort (Northam et al., 2012b), here we explored the relationship between language lateralization during a verb generation task (Northam et al., 2012b) and oromotor skills in the subgroup who had both fMRI and diffusion MRI data.

## Materials and Methods

### Participants

Thirty-six adolescents (13 males, mean age 16 ± 1 years) born preterm (mean gestation age 27 weeks, range 26–31; mean birthweight 1060 g, range 591 g to 2243 g) out of a cohort of 50 as part of a previous study (Northam et al., 2012a) had undergone (i) a speech and oromotor assessment and (ii) MRI scanning including a high angular resolution diffusion imaging (HARDI). Of this sample, 25 had injury on cranial ultrasound at birth (15 minor, 10 major) and four had cerebral palsy (one bilateral, three predominantly unilateral).

Ethical approval for the study was obtained from Great Ormond Street Hospital for Children/Institute of Child Health Research Ethics Committee. All parents or participants gave written informed consent.

### Definition of Oromotor Impairment

Eleven of the 36 adolescents were previously classified as having an oromotor impairment (two mild-moderate, and nine severe who scored 60–91%) on the Focal Oromotor Control (FOC) subtest from the Verbal Motor Production Assessment for Children (Hayden and Square, 1999). They, however, showed no impairment in speech sound production during everyday speech warranting a diagnosis of motor speech disorder. This was assessed using the Connected Speech and Language subtest, where motor precision is evaluated during the spontaneous description of a story based on four sequenced pictures. The VMPAC is a standardized tool which assesses neuromuscular control of the articulators during speech and non-speech (“oromotor”) movements with high reliability. The FOC subtest assesses deliberate control of the jaw, lips, face, and tongue. Participants are required to execute movements (e.g., tongue protrusion, smiling, blowing) and speech sounds in isolation and in sequence (e.g., /a/ /m/ /u/).

The VMPAC was administered by a trained administrator (GN) at the time of MRI acquisition according to the specific guidelines established in the video accompanying the VMPAC manual. GN and AM had three training sessions to confirm correct administration. Following training, each testing session was video recorded. These recordings were rated by a speech and language pathologist (AM) with over 20 years’ experience in the field of differential diagnosis of motor speech disorders, including in children born pre-term and numerous other neurological and neurodevelopmental populations. The assessment was rated for impaired facial symmetry, tone, and smoothness of movement.

### Diffusion MRI

An eddy-current-nulled twice-refocused EPI sequence with high-angular resolution was acquired (*b*-value = 3000 s/mm^2^, TE = 128 ms, 60 diffusion-weighted directions, in-plane resolution 2.1 mm × 2.1 mm, 3 mm slice thickness, 37 contiguous axial slices, acquisition time ∼9 min). Further diffusion imaging protocol details can be found elsewhere (Northam et al., 2012a,b).

### Tractography of the Corticobulbar and Corticospinal Tracts

**FIGURE 1:**
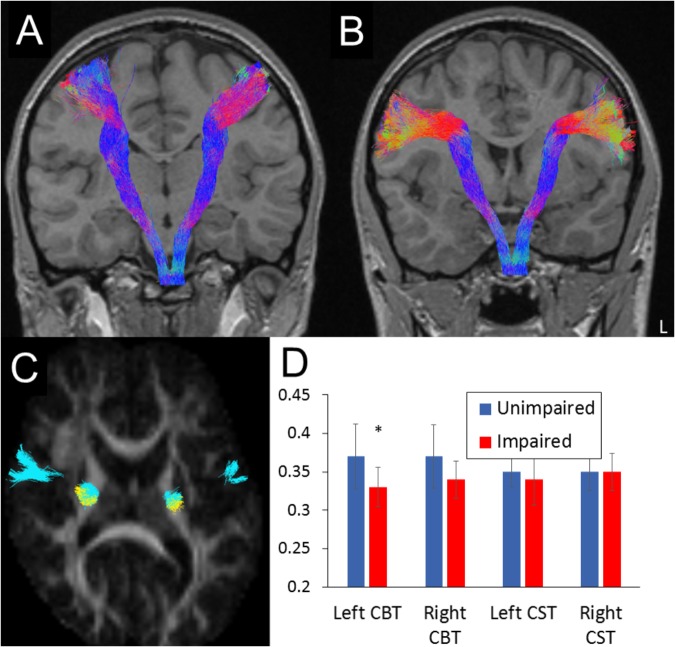
Illustrative examples of **(A)** corticospinal and **(B)** dorsal corticobulbar tract tractography reconstruction in a participant. Tracts are projected on a T1-weighted MRI scan in coronal plane to allow view along the full tract length. Axial cross section **(C)** illustrates partial overlap of the two tracts (blue, corticobulbar or CBT; yellow, corticospinal or CST) at the level of the posterior limb of the internal capsule. L, left hemisphere. **(D)** Group differences in mean FA for each tract (error bars represent SDs; ^∗^ statistical difference *p* < 0.01).

DWI datasets were pre-processed using the MRTrix software suite (Tournier et al., 2012). Tractography methods were as described in Liegeois et al. (2013) and performed in native space using a probabilistic streamlines algorithm^1^. Two components of the corticobulbar tract were tracked using spherical seed ROIs placed in the axial plane (radius 7 mm). The seed region for the CST was centered in the white matter adjacent to the precentral gyrus (at the level of the hand “omega”). The ROI for the dorsal corticobulbar tract was placed 15 mm inferior to the hand area, and the ventral corticobulbar tract was centered another 15 mm inferiorly. For all three tracks, an inclusion ROI was manually placed at the level of the pons (Liegeois et al., 2013, see Supplementary Figure S1A). We set the maximum number of streamlines generated at 100,000, with a maximum of 1,000 streamlines retained. A binary mask of all voxels containing selected streamlines was created for each of the six tracks. Mean FA was extracted within each mask. An example case is shown in Figure 1A–C.

### Language Lateralization on fMRI

Details of the fMRI protocol are provided elsewhere (Northam et al., 2012b). Briefly, participants were asked to generate single verbs, without vocalization, associated with single words presented via earphones (task condition). Two runs of ten task/rest cycles were completed by each participant. Lateralization indices (LI) were calculated within defined regions of interest, including an extended Broca’s area (pars triangularis and opercularis, premotor cortex, and middle frontal gyrus), the temporal lobes (inferior, middle, and superior gyri combined) and the cerebellum. As fMRI-derived lateralization indices are threshold dependent, they were calculated at multiple statistical thresholds using a bootstrapping method as implemented in the LI-toolbox (Wilke and Lidzba, 2007). Indices range from +1 (complete left lateralization) to -1 (complete right lateralization). Atypical lateralization in Broca’s region was defined by a LI value <0.2 (Pahs et al., 2013).

### Statistical Analyses

Fractional anisotropy values were compared between groups using univariate analyses of covariance (age as a covariate). Spearman’s correlations were used to assess the relationship between track measures and oromotor scores. LI were compared between impairment groups using independent sample *t*-tests. A binary logistic regression was computed to identify imaging predictors of oromotor impairment.

## Results

### Clinical and Neuropsychological Characteristics

Participants with focal oromotor control (FOC) impairment had greater degree of neurological and imaging abnormalities than those without impairment (see Supplementary Table S1), together with lower IQ and language test scores.

### Group Differences in Motor Tracts

The oromotor-impaired group showed reduced FA within the left-dorsal CBT compared to the unimpaired group (Figure 1D) [*F*(1,30) = 7.97, *p* = 0.009; 95% confidence interval (CI) of the mean difference 0.012–0.073; partial eta squared = 0.215). This reduction remained significant when controlling for full scale IQ (*F* = 5.47, *p* = 0.027, partial eta squared = 0.17] or CELF language scores (*F* = 5.22, *p* = 0.03, partial eta squared = 0.157). No other tract differences were found (all *p* > 0.07), although the reduction in the left ventral CBT approached significance (*p* = 0.074). Mean FA from within the left dorsal (ρ = 0.54, *p* = 0.001, see Supplementary Figure S1C) and ventral (ρ = 0.36, *p* = 0.041) CBT correlated positively with scores on the FOC subtest across the whole sample.

To determine the most robust predictors of oromotor impairment, we entered the following predictor variables into a forward binary logistic regression: abnormal neurological examination, abnormality on conventional MRI, mean FA from left posterior limb of the internal capsule (as used previously, Northam et al., 2012a) and FA in left-dorsal CBT. A test of the full model against the constant revealed that the only significant independent predictor of FOC impairment was mean FA in the left-dorsal CBT (*B* = -39.43, *SE* = 16.72; Exp(B) < 0.0001, 95% CI for Exp(B) = <0.001 to 0.001), correctly classifying 75% of the sample. The final model was statistically significant (Chi Square = 55.59, *p* = 0.018), and explained a large proportion of variance (Nagelkerke *R* = 0.34). Removal of the term led to a significant change in -2 log likelihood ratio (*p* = 0.002). Given the small number of participants with impairments, these results have to be interpreted with caution.

### fMRI Language Lateralization

**FIGURE 2:**
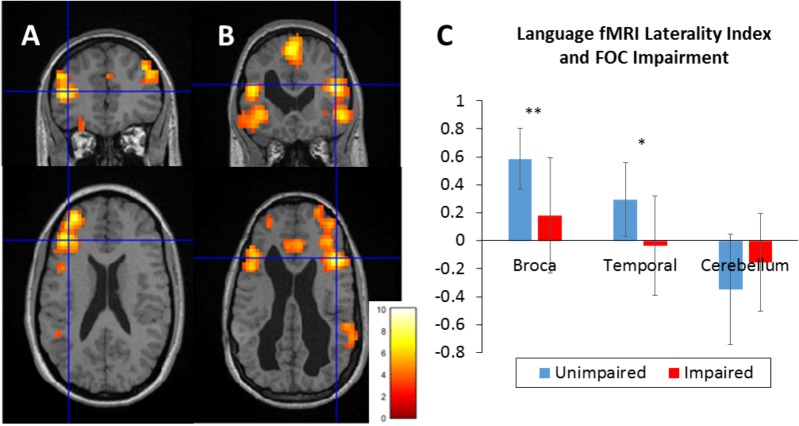
fMRI language laterality in two example cases **(A)** – without oromotor impairment, **(B)** – with oromotor impairment, and **(C)** mean laterality indices in focal oromotor impairment groups. Both cases had sustained major preterm birth-related brain injury (still visible as enlargements of the lateral ventricles). Maps represent single subject activation during a verb generation task (thresholded at *p* < 0.001, color bar shows *z*-values). Left hemisphere is displayed on the left. Crosshair indicates inferior frontal activation peak. In case **(A)** with bilateral hemorrhagic lesion in the frontal cortex (left < right) shows left lateralization (Broca’s laterality index = +0.6). In case **(B)** (slurred speech at 4 years, now resolved) with bilateral hemorrhagic lesion in the frontal cortex (left > right) language was represented bilaterally (laterality index = –0.1). **(C)** Group differences in laterality indices in regions of interest (Broca’s region, temporal lobe, and cerebellum). Error bars represent SD; statistical difference: ^∗∗^*p* < 0.001, ^∗^*p* < 0.01.

In the preterm group, activation for the generate > listen contrast was detected (random effect analysis, *P* < 0.05, corrected for multiple comparisons) in the left inferior frontal regions extending into the precentral and middle frontal gyrus, the superior temporal gyrus, and the right cerebellum (see Supplementary Figure S1B). Two example cases of children with major bilateral brain injury (hemorrhagic parenchymal infarction grade 4) are shown in Figure 2. Case A with intact oromotor function shows typical left-sided lateralization, as seen in 88% of this group. In contrast, case B with oromotor impairment shows strong recruitment of the right inferior frontal and temporal cortices, seen in 64% of this group (Fisher’s exact: *p* = 0.003). This pattern is also reflected in the mean LI (Figure 2C), showing lower values in Broca’s region (*t*_df=34_ = 3.91, *p* < 0.001) and temporal lobes (*t*_df=34_ = 3.10, *p* = 0.004), but not the cerebellum (*p* = 0.152) in those with oromotor impairment relative to those without. There was a positive correlation between FA in the left CBT (dorsal and ventral combined) and LI values derived from Broca’s region (ρ = 0.383, *p* = 0.030).

## Discussion

Using DWI-tractography in a group of adolescents born preterm, we have identified an association between subclinical oromotor impairment and the microstructural integrity of the primary motor pathway. We focused on the dorsal component of the corticobulbar tract, as this is known to originate from the primary motor representations of the larynx, lips, jaw, and tongue (Takai et al., 2010; Liegeois et al., 2016). This anatomically constrained approach provided a much better predictor of oromotor problems than less specific measures of internal capsule integrity (shown previously, Northam et al., 2012a) in line with other studies indicating a relationship between limb motor outcome and the microstructure of motor tracts in preterm individuals (Groeschel et al., 2014).

We also demonstrated (using an fMRI language task) that the presence of oromotor impairment and left corticobulbar tract injury is associated with greater recruitment of Broca’s homolog in the right hemisphere. Moreover, we identified a positive relationship between the severity of left corticobulbar tract damage and the degree of language reorganization. The apparent reorganization of both motor speech and language regions to the right hemisphere suggests functional co-dependence between the two during language development. However, we cannot exclude that prenatal brain injury affected both systems in the left hemisphere, given their anatomical proximity.

Our findings agree with previous studies suggesting that early periventricular injury is associated with atypical language representation (Staudt et al., 2001). In those children with persistent oromotor impairments and atypical speech development, we would interpret this as a compensatory mechanism. In support of this view, inter-hemispheric plasticity has been shown to diminish the behavioral impact of lesions on language development in children following neonatal stroke (Northam et al., 2018).

Importantly, the potential for interhemispheric plasticity following brain injury appears to depend on the timing of the lesion. For instance, children with dysarthria following traumatic left corticobulbar tract damage have been found to have typical left hemisphere language lateralization (Morgan et al., 2013). The later timing of the injury in these children (from 3 to 16 years) may account for the lack of interhemispheric reorganization, as the potential for structural and functional plasticity is maximal in infancy. This view is also in keeping with recent evidence in childhood stroke, showing poorer language outcomes and less right hemisphere reorganization for injuries occurring after the age of 5 years (Ilves et al., 2014; Lidzba et al., 2017a,b).

Although we have identified clear evidence of oromotor impairments in the preterm group – for instance when performing sequential kissing and smiling movements or producing randomly arranged speech sounds (e.g., /a//m//u/) – we describe this as “subclinical” since these unusual combinations do not occur frequently in everyday speech. However, what is unclear is the developmental impact of such subtle speech-motor difficulties in preterm children. Although the problems in our cohort were modest at the time of assessment (adolescence), it has been suggested that there may be a link between oromotor control problems and disruption of early language development (Alcock, 2006; Krishnan et al., 2013). For instance, FA differences have been identified in the left corticobulbar tract in children with developmental speech disorders of unknown origin (Morgan et al., 2018). Although in our cohort, detailed speech, and language assessments were not undertaken in childhood, parental reports indicate that impaired participants were more likely to have received speech and language therapy – or to have displayed some form of atypical speech development (e.g., late onset, poor speech quality/tongue control; Northam et al., 2012a). It seems plausible that such problems might lead to “knock-on” effects on early cognitive development, education, and subsequent school performance. There may therefore be an argument for early identification of these children to enable timely intervention. Overall, our findings highlight the need for a prospective longitudinal study examining early speech and oromotor development in infants born preterm, with and without corticobulbar tract injury.

In conclusion, we provide further evidence (see also Liegeois et al., 2013) that early injury to the left corticobulbar tract is detrimental to the development of oromotor control. We have also shown that despite apparently normal everyday speech, persistent deficits are nevertheless detectable many years later during novel oromotor tasks. Moreover, we have demonstrated recruitment of the right hemisphere language areas in these individuals – and suggest that this may be a compensatory mechanism contributing to the favorable long-term speech outcomes in preterm children.

## Data Availability Statement

The datasets for this manuscript are not publicly available because participants and guardians have not given consent for data sharing. Requests to access the datasets should be directed to TB (t.baldeweg@ucl.ac.uk).

## Author Contributions

GN contributed to study design, acquired and analyzed the data, and wrote the manuscript. AM contributed to study concept and design, study supervision, provided speech/oromotor diagnosis, and critically revised the manuscript for intellectual content. SF performed the tractography analysis. TB contributed to study concept and design, study supervision, data analysis, and critically revised of manuscript for intellectual content. FL contributed to study concept and design, analysis of data, study supervision, and wrote the manuscript.

## Conflict of Interest Statement

The authors declare that the research was conducted in the absence of any commercial or financial relationships that could be construed as a potential conflict of interest.

## Footnotes

1 http://www.mrtrix.org/

## Supplementary Material

FIGURE S1
**(A)** Seed spherical regions (7 mm radius) of interest and target pons region (yellow) used for the tractography reconstructions of the motor tracts (see Liegeois et al., 2013, for details). The center of the spherical seed for the corticospinal tract is placed on the precentral white matter in the “hand knob.” The centers of the dorsal and ventral corticobulbar seed spheres are located 15 and 30 mm ventrally to the hand center, but more anteriorly to follow the course of the precentral white matter. The target inclusion region in the pons (in yellow on the sagittal and coronal views of color coded FA maps, see arrows) is delineated on an axial slice to ensure it covers the cross section of the pyramidal tract. **(B)** Functional MRI activation for the Generate > Listen contrast (covertly generating single verbs vs. listening to bursts of amplitude-modulated white noise) in the preterm group. Results are projected onto an inflated single subject template (dspmview toolbox) at *p* = 0.05, family-wise error (FWE) correction. The color bar indicates *T*-value. Left hemisphere is on the left. Activated clusters included the left inferior frontal region (peak at -38, 22, 2) extending into the precentral and middle frontal gyri, the supplementary motor area and anterior cingulate cortex (peak at -6, 4, 56), the right cerebellum (peak at 38, -66, 30), left (peak at -58, -22, 4), and right superior temporal gyri (peak at 60, -32, 6) as well as the right anterior insular/opercular cortex (peak 40, 20, 2). **(C)** Relationship between focal oromotor scores and corticobulbar tract FA in the whole group.

TABLE S1 Clinical and neuropsychological characteristics of focal oromotor impairment groups.
