# Supplementary figures and images for: Corticobulbar Tract Injury, Oromotor Impairment and Language Plasticity in Adolescents Born Preterm
Source: Front Hum Neurosci. 2019 Feb 19;13:45. doi: 10.3389/fnhum.2019.00045 (PMC6389783; doi:10.3389/fnhum.2019.00045)

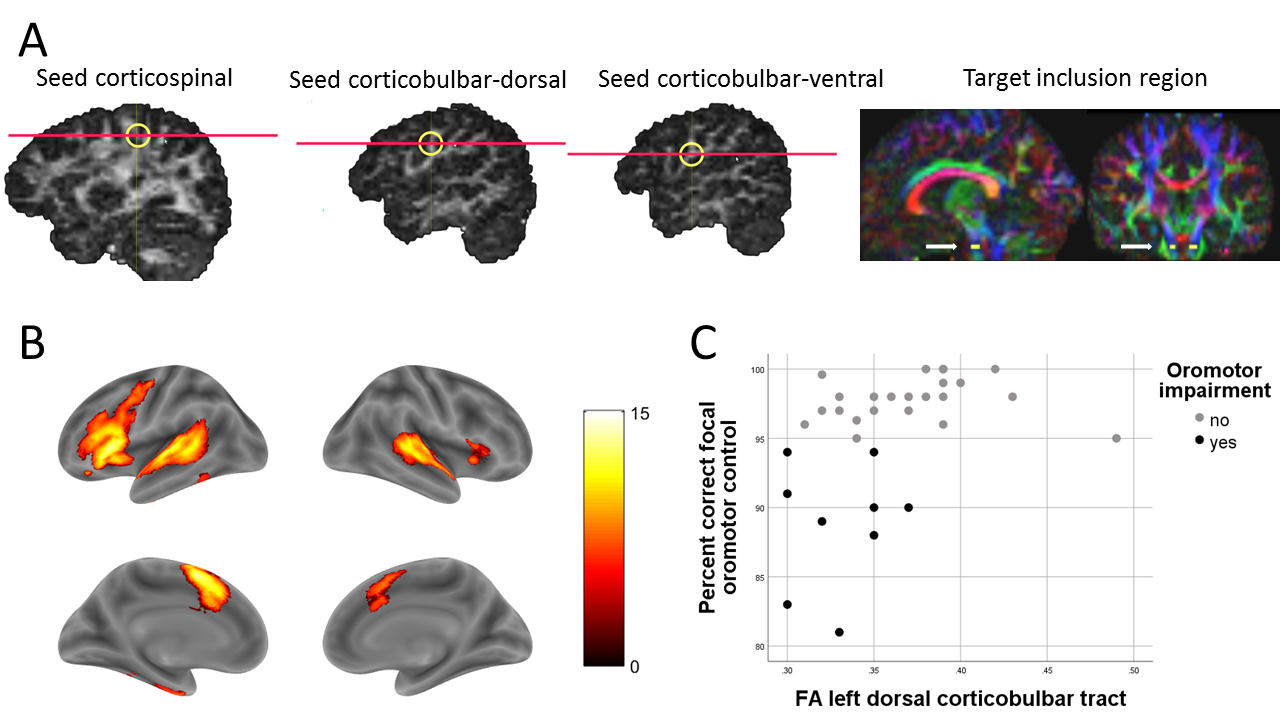

Supplement: FIGURE S1 — (A) Seed spherical regions (7 mm radius) of interest and target pons region (yellow) used for the tractography reconstructions of the motor tracts (see Liegeois et al., 2013, for details). The center of the spherical seed for the corticospinal tract is placed on the precentral white matter in the “hand knob.” The centers of the dorsal and ventral corticobulbar seed spheres are located 15 and 30 mm ventrally to the hand center, but more anteriorly to follow the course of the precentral white matter. The target inclusion region in the pons (in yellow on the sagittal and coronal views of color coded FA maps, see arrows) is delineated on an axial slice to ensure it covers the cross section of the pyramidal tract. (B) Functional MRI activation for the Generate > Listen contrast (covertly generating single verbs vs. listening to bursts of amplitude-modulated white noise) in the preterm group. Results are projected onto an inflated single subject template (dspmview toolbox) at p = 0.05, family-wise error (FWE) correction. The color bar indicates T-value. Left hemisphere is on the left. Activated clusters included the left inferior frontal region (peak at -38, 22, 2) extending into the precentral and middle frontal gyri, the supplementary motor area and anterior cingulate cortex (peak at -6, 4, 56), the right cerebellum (peak at 38, -66, 30), left (peak at -58, -22, 4), and right superior temporal gyri (peak at 60, -32, 6) as well as the right anterior insular/opercular cortex (peak 40, 20, 2). (C) Relationship between focal oromotor scores and corticobulbar tract FA in the whole group. [file Image_1.TIF]
