# Supplementary material for: Corticobulbar Tract Injury, Oromotor Impairment and Language Plasticity in Adolescents Born Preterm
Source: Front Hum Neurosci. 2019 Feb 19;13:45. doi: 10.3389/fnhum.2019.00045 (PMC6389783; doi:10.3389/fnhum.2019.00045)
Supplement: TABLE S1 — Clinical and neuropsychological characteristics of focal oromotor impairment groups. [file Table_1.docx]

**Supplementary Table 1** Clinical and neuropsychological characteristics of focal oromotor impairment groups.

|  | Unimpaired focal  oromotor (n = 25) | Impaired focal  oromotor (n = 11) | Statistical  comparison |
| --- | --- | --- | --- |
| Cranial ultrasound findings at birth | 18 (72%) | 7 (64%) | P = .45 |
| Neurologic outcome  abnormal | 7 (28%) | 7 (64%) | P = .05 |
| MRI abnormalities | 17 (68%) | 11 (100%) | P = .04 |
| Previous speech and  language therapy | 5 (20%) | 6 (55%) | P = .05 |
| Mean full-scale IQ (SD) | 95 (13) | 76 (14) | P < .001 |
| Mean language (CELF) total score (SD) | 91 (18) | 71 (14) | P = .001 |

NB. **'Neurologic outcome abnormal'** refers to the outcome of a standardised neurological examination and includes major (e.g. cerebral palsy) or minor abnormalities (e.g. asymmetry of reflexes, hypotonia).**'MRI abnormalities':** All scans were evaluated by an experienced pediatric neuroradiologist and refers to findings considered to be consistent with preterm birth (see Northam et al. 2011).
